# Testing for pharmacogenomic predictors of ppRNFL thinning in individuals exposed to vigabatrin

**DOI:** 10.3389/fnins.2023.1156362

**Published:** 2023-09-08

**Authors:** Isabelle Boothman, Lisa M. Clayton, Mark McCormack, Alexandra McKibben Driscoll, Remi Stevelink, Patrick Moloney, Roland Krause, Wolfram S. Kunz, Sarah Diehl, Terence J. O’Brien, Graeme J. Sills, Gerrit-Jan de Haan, Federico Zara, Bobby P. Koeleman, Chantal Depondt, Anthony G. Marson, Hreinn Stefansson, Kari Stefansson, John Craig, Michael R. Johnson, Pasquale Striano, Holger Lerche, Simon J. Furney, Norman Delanty, Consortium EpiPGX, Joseph Willis, Mojgansadat Borghei, Simona Donatello, Martin J. Brodie, Pauls Auce, Andrea Jorgensen, Sarah R. Langley, Yvonne Weber, Christian Hengsbach, Martin Krenn, Fritz Zimprich, Ekaterina Pataraia, Karl Martin Klein, Hiltrud Muhle, Rikke S. Møller, Marina Nikanorova, Stefan Wolking, Ellen Campbell, Antonella Riva, Marcello Scala, Sanjay M. Sisodiya, Gianpiero L. Cavalleri

**Affiliations:** ^1^School of Pharmacy and Biomolecular Sciences, Royal College of Surgeons in Ireland, Dublin, Ireland; ^2^The SFI Futureneuro Research Centre, Royal College of Surgeons in Ireland, Dublin, Ireland; ^3^The SFI Centre for Research Training in Genomics Data Science, Galway, Ireland; ^4^Department of Clinical and Experimental Epilepsy, UCL Queen Square Institute of Neurology, London, United Kingdom; ^5^Chalfont Centre for Epilepsy, Bucks, United Kingdom; ^6^Department of Genetics, University Medical Center Utrecht, Utrecht, Netherlands; ^7^Luxembourg Centre for Systems Biomedicine, University of Luxembourg, Esch-sur-Alzette, Luxembourg; ^8^Division of Neurochemistry, Department of Epileptology, University Bonn Medical Center, Bonn, Germany; ^9^Departments of Neuroscience and Neurology, Central Clinical School, The Alfred Hospital, Monash University, Melbourne, VIC, Australia; ^10^School of Life Sciences, University of Glasgow, Glasgow, United Kingdom; ^11^Stichting Epilepsie Instellingen Nederland (SEIN), Heemstede, Netherlands; ^12^"IRCCS”G. Gaslini" Institute, Genova, Italy; ^13^Department of Neurosciences, Rehabilitation, Ophthalmology, Genetics, Maternal and Child Health, University of Genoa, Genova, Italy; ^14^Department of Neurology, Hôpital Erasme, Hôpital Universitaire de Bruxelles, Université Libre de Bruxelles, Brussels, Belgium; ^15^Department of Pharmacology and Therapeutics, University of Liverpool, Liverpool, United Kingdom; ^16^deCODE Genetics, Reykjavik, Iceland; ^17^Department of Neurology, Royal Victoria Hospital, Belfast Health and Social Care Trust, Belfast, United Kingdom; ^18^Division of Brain Sciences, Imperial College Faculty of Medicine, London, United Kingdom; ^19^Department of Neurology and Epileptology, Hertie Institute for Clinical Brain Research, University of Tübingen, Tübingen, Germany; ^20^Genomic Oncology Research Group, Deptartment of Physiology and Medical Physics, Royal College of Surgeons in Ireland (RCSI), Dublin, Ireland

**Keywords:** adverse drug reaction, epilepsy, retina, genome wide association study, polygenic risk score

## Abstract

**Background:**

The anti-seizure medication vigabatrin (VGB) is effective for controlling seizures, especially infantile spasms. However, use is limited by VGB-associated visual field loss (VAVFL). The mechanisms by which VGB causes VAVFL remains unknown. Average peripapillary retinal nerve fibre layer (ppRNFL) thickness correlates with the degree of visual field loss (measured by mean radial degrees). Duration of VGB exposure, maximum daily VGB dose, and male sex are associated with ppRNFL thinning. Here we test the hypothesis that common genetic variation is a predictor of ppRNFL thinning in VGB exposed individuals. Identifying pharmacogenomic predictors of ppRNFL thinning in VGB exposed individuals could potentially enable safe prescribing of VGB and broader use of a highly effective drug.

**Methods:**

Optical coherence topography (OCT) and GWAS data were processed from VGB-exposed individuals (*n* = 71) recruited through the EpiPGX Consortium. We conducted quantitative GWAS analyses for the following OCT measurements: (1) average ppRNFL, (2) inferior quadrant, (3) nasal quadrant, (4) superior quadrant, (5) temporal quadrant, (6) inferior nasal sector, (7) nasal inferior sector, (8) superior nasal sector, and (9) nasal superior sector. Using the summary statistics from the GWAS analyses we conducted gene-based testing using VEGAS2. We conducted nine different PRS analyses using the OCT measurements. To determine if VGB-exposed individuals were predisposed to having a thinner RNFL, we calculated their polygenic burden for retinal thickness. PRS alleles for retinal thickness were calculated using published summary statistics from a large-scale GWAS of inner retinal morphology using the OCT images of UK Biobank participants.

**Results:**

The GWAS analyses did not identify a significant association after correction for multiple testing. Similarly, the gene-based and PRS analyses did not reveal a significant association that survived multiple testing.

**Conclusion:**

We set out to identify common genetic predictors for VGB induced ppRNFL thinning. Results suggest that large-effect common genetic predictors are unlikely to exist for ppRNFL thinning (as a marker of VAVFL). Sample size was a limitation of this study. However, further recruitment is a challenge as VGB is rarely used today because of this adverse reaction. Rare variants may be predictors of this adverse drug reaction and were not studied here.

## 1. Introduction

The anti-seizure medication (ASM) vigabatrin (VGB) was first licensed in 1989 as an adjunctive therapy for individuals with focal seizures (Russell-Eggitt et al., 2000). VGB irreversibly inhibits GABA transaminase leading to increased intracellular concentrations of GABA, a major neurotransmitter in inhibitory central nervous system pathways (Jacob et al., 1990; Davies, 1995; Ben-Menachem, 2011).

The use of VGB, however, is limited by the risk of vigabatrin-associated visual field loss (VAVFL). First reported in 1997 (Eke et al., 1997), VAVFL has been shown to effect up to 44% of VGB-exposed adults and 29% of infants (Lawden et al., 1999; Maguire, 2010; Biswas et al., 2020), and is characterized by irreversible concentric peripheral field loss with temporal and macular sparing (Wild et al., 1999). VAVFL is usually assessed using perimetry (Nousiainen et al., 2001; Paul et al., 2001; Clayton et al., 2013), which has inherent limitations (Clayton et al., 2013). Optical coherence topography (OCT) provides a quantification of peripapillary retinal nerve fibre layer (ppRNFL) thickness that has been shown to correlate strongly with visual field size in people with VAVFL, and is easier and more reliable to undertake (Lawthom et al., 2009; Clayton et al., 2011; Moseng et al., 2011; Kjellström et al., 2014).

The correlation between visual field size and ppRNFL thickness in individuals with VAVFL has led to the suggestion that retinal ganglion cell (RGC) loss may contribute to the retinal pathology leading to visual dysfunction in VAVFL. Furthermore, in VGB-exposed individuals OCT-quantified ppRNFL loss was most frequently observed in the superior and inferior quadrants (Clayton et al., 2011), with early involvement of the nasal superior sector (Clayton et al., 2012), while the temporal region appeared unaffected (Clayton et al., 2011, 2012), suggesting that certain populations of RGC may be more vulnerable (Clayton et al., 2012). However, there is no clear evidence as to whether RGCs are the primary target for VGB toxicity, or whether RGC loss occurs secondary to other retinal cell pathology (Clayton et al., 2011), and the exact mechanism by which VGB causes VAVFL remains unknown (Heim and Gidal, 2012). Studies in animal models have shown that VGB damages the cone photoreceptors, bipolar cells, and the retinal ganglion cells (Duboc et al., 2004; Wang et al., 2008; Jammoul et al., 2010; Chan et al., 2020) as well as driving changes in mitochondria (Vogel et al., 2017). Animal models exposed to VGB display an increased concentration of GABA in the retina (Yee et al., 1998; Chan et al., 2020).

As a result of VAVFL, licensing authorities have restricted the use of VGB. Today, VGB is licensed for use as an adjunctive therapy in focal epilepsy where other drugs have failed, and where the benefits of the treatment outweigh the risk of VAVFL, and as a monotherapy in the treatment of infantile spasms (EMA, 2018; Bresnahan et al., 2020; FDA, 2020). In individuals with infantile spasms particularly those with tuberous sclerosis complex (TSC), studies have shown that VGB is more effective than hormone therapy and steroids and should be used as the first drug to treat this condition (Chiron et al., 1997; Vigevano and Cilio, 1997; Hancock and Osborne, 1999; Chiron, 2016; Messer and Knupp, 2020; Schubert-Bast and Strzelczyk, 2021). Identifying genetic predictors of VAVFL could potentially enable safe prescribing of VGB and broader use of an otherwise highly effective medication. Previous studies have been unsuccessful in identifying genetic predictors of VAVFL; a candidate gene approach correlating VAVFL and genetic variation across six candidate genes (*SLC6A1, SLC6A13, SCL6A11, ABAT, GABRR1,* and *GABRR2*) found three significant associations between single tagging SNPS and visual field size. However, these findings did not replicate in an independent cohort (Kinirons et al., 2006). Another candidate gene approach, focused on ornithine-aminotransferase, did not find clinically significant genetic variation relevant to VAVFL (Hisama et al., 2001).

In this study we set out to identify genetic predictors of ppRNFL thinning in VGB exposed individuals. This aim was supported by the objectives of (1) conducting a quantitative GWAS of OCT measurements in people exposed to VGB, (2) identifying genetic predictors of ppRNFL thinning in VGB-exposed individuals using gene-based analysis of GWAS summary statistics and (3) determining whether individuals exposed to VGB have a polygenic burden for a thinner retinal thickness using polygenic risk scoring (PRS) analysis.

## 2. Methods

All participants (or their legal guardians in the case of individuals with intellectual disability) provided written, informed consent for this study. Ethical approval was provided by the relevant ethics boards at each study site; Beaumont Hospital (study code 14/44). The University College London (UCL) Queen Square Institute of Neurology (study code 11/LO/2016) and the University Medical Centre, Utrecht (study codes 09/352 and 18–466).

### 2.1. Cohort and data description

We studied samples from the EpiPGX Consortium, contributed from the following three sites: the Royal College of Surgeons in Ireland (RCSI, Dublin, Ireland), University College London (UCL) Queen Square Institute of Neurology (London, United Kingdom), and the University Medical Centre (Utrecht, Netherlands). To be included in the study, individuals had to have been exposed to VGB and had OCT performed (post drug exposure) using a standard protocol (see optical coherence tomography methods below). Where VAVFL was present, it must have led to withdrawal or dose reduction of VGB, and not be attributed to another cause by treating clinicians or the phenotyping clinician. Previous brain surgery for epilepsy was an exclusion criterion, given surgery can lead to visual defects which would confound the results (Marino and Rasmussen, 1968; van Lanen et al., 2018).

### 2.2. Optical coherence tomography

All research participants underwent ppRNFL imaging using spectral-domain optical coherence tomography (Cirrus HD_OCT, software version 5.0 and 7.01.290; Carl Zeiss Meditec, Dublin, CA). The optic disc cube 200×200 protocol was used to measure ppRNFL thickness. This protocol has a 6×60-mm grid of data generated by acquiring 200 horizontal scans which are composed of 200 A-scans centred over the optic disc. The glaucoma analysis algorithm was used to measure ppRNFL thickness. When using this algorithm, a 3.46-mm diameter circle of data made up from 256 A-scans is used to measure ppRNFL thickness (Clayton et al., 2012).

We used nine different quantitative OCT thickness measurements in our analysis: (1) average retinal nerve fiber layer, (2) inferior quadrant, (3) nasal quadrant, (4) superior quadrant, (5) temporal quadrant, (6) inferior nasal sector, (7) nasal inferior sector, (8) superior nasal sector and (9) nasal superior sector, according to published methods (Clayton et al., 2011).

### 2.3. Imputation and quality control

DNA from study participants were genotyped using a combination of Illumina (San Diego, CA) OmniExpress-12 v1.1 and OmniExpress-24 v1.1 single nucleotide polymorphism (SNP) arrays. Imputation and pre imputation quality control processes were performed as detailed elsewhere (McCormack et al., 2018).

After imputation and merging of the samples, samples with >90% call rate, SNPs with >90% INFO score, >95% call-rate, MAFs >1% and HWE deviations *p* > 1e-6 were kept. To ensure genetic homogeneity within the analytic dataset, the top two genetic principal components (PCs) were calculated using PLINK (Purcell et al., 2007) and plotted using ggplot2 (Wickham, 2016). Any outliers on the PCA plot were removed from further analysis.

We ran power calculations to determine the study’s statistical power in R, using a previously published protocol developed for quantitative traits (R Core Team, 2022).

### 2.4. GWAS and univariate analysis

Quantitative GWAS analyses were conducted on the 9 quantitative OCT measurements (see optical coherence tomography section above) using SNPTEST and applying the ‘em’ model (Marchini et al., 2007). Sex, cumulative dose, maximum daily dose, and duration of prescription of VGB (years) were included as covariates in the SNPTEST model as these factors have been reported to be correlated with ppRNFL thinning (Clayton et al., 2012). We also included as covariates (in SNPTEST) the first 4 PCs from PCA analysis (see Imputation and quality control section above) to control for population stratification. Manhattan and quantile-quantile plots (QQ plots) were generated for each analysis using the R package qqman (Turner, 2018).

### 2.5. Gene-based testing

Gene-based testing (via VEGAS2 (Mishra and Macgregor, 2015)) was used to identify genes containing multiple risk variants that individually are weakly associated with a univariate trait. VEGAS2 works by first assigning SNPs to genes based on the genomic location and then calculating gene-based empirical association *p*-values (Liu et al., 2010; Mishra and Macgregor, 2015). Briefly, for a given gene with n SNPs, single SNP association *p* values are first converted to upper-tail chi-squared statistics with one degree of freedom (df). The gene-based test statistic is the sum of all (or a pre-defined subset) of the chi-squared 1 df statistics within that gene (Liu et al., 2010; Mishra and Macgregor, 2015).

In each analysis, VEGAS2 assigned 5,954,017 SNPs to 20,489 genes. We set the gene boundaries to include Intergenic SNPs in high linkage disequilibrium (*r*^2^ > 0.8) with SNPs within a gene (0kbldbin). We used the European reference panel in the VEGAS2 analysis. We applied Bonferroni correction to control for multiple testing, with the threshold for significance set *p* < 2.71 × 10^−7^ (i.e., 0.05/(no. of genes X no. of OCT measurements)).

We cross referenced results of our gene-based testing with 26 genes with ocular function that were previously shown to be differentially expressed in mice exposed to VGB, compared to the controls (Walters et al., 2020).

### 2.6. Polygenic risk scoring

PRS alleles for retinal thickness were calculated using the summary statistics from a GWAS of inner retinal morphology using OCT images of 31,434 UK Biobank participants (Currant et al., 2021). We conducted individual PRS analyses for each of the nine OCT measurements detailed above.

Eight p value thresholds (0.01, 0.05, 0.1, 0.2, 0.3, 0.4, 0.5, and 1) were used to select PRS alleles. We corrected for multiple testing using Bonferroni correction by multiplying the results by the number of thresholds and by the number of OCT measurements. Statistical analyses of the data were carried out in R.4.0.2 (R Core Team, 2022).

We included the following covariates in the PRS analysis; sex, cumulative dose, maximum daily dose, duration of prescription of VGB (years) and 4 PCs. We used PRSice-2 to calculate the risk scores and to generate a linear regression model and estimate *β*-coefficients and standard errors for each PRS analysis (Choi and O’Reilly, 2019).

**Figure 1:**
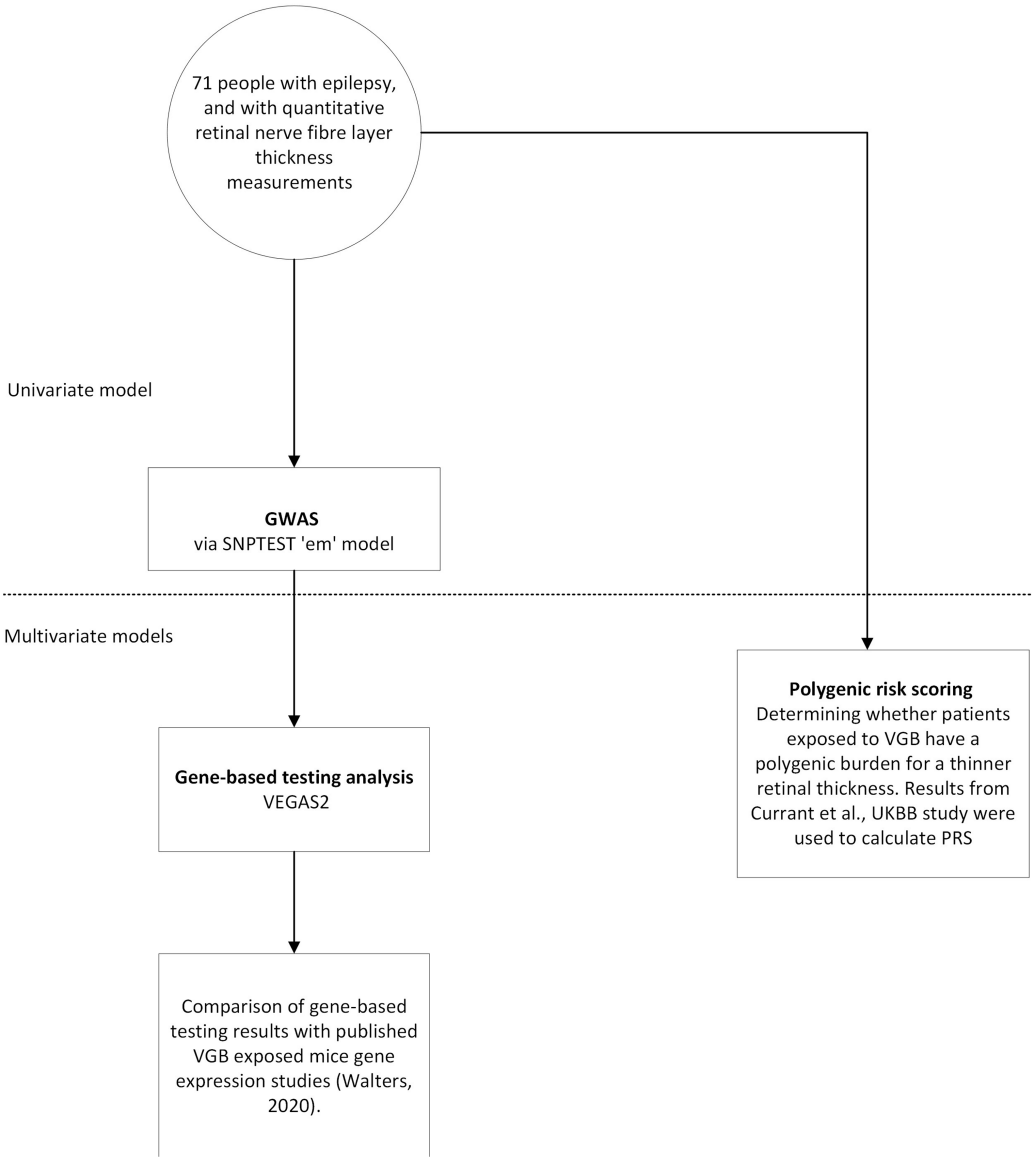
Schematic diagram of the methodology.

Schematic diagram of methods is shown in Figure 1.

## 3. Results

### 3.1. Cohort

The study cohort consisted of 140 people with epilepsy exposed to VGB. After excluding individuals that had epilepsy surgery and completing QC, 71 individuals were brought forward for the GWAS. A description of the cohort is provided in Table 1.

**Table 1:** Patient cohort description.

Sex (Male/Female)	59/49
Age (years)	68.35 (40–96)
Duration (years)	4.86 (0.083–22.75)
Daily max dose (g)	2.28 (0–4)
Cumulative dose (g)	3406.1 (14–20,085)

Description of patient cohort (*n* = 108). Description of the cohort details the number of males and females, the average age of the cohort, the duration of VGB prescription (years), the daily maximum dose of VGB (g) and the cumulative dose of VGB (g).

### 3.2. GWAS

To identify univariate, common genetic predictors of ppRNFL thinning (as a marker of VAVFL) in VGB-exposed individuals we conducted GWAS of nine quantitative OCT measurements of ppRNFL with the cohort of 71 VGB-exposed individuals. No single variant reached the threshold for genome-wide significance (5 × 10^−8^). We detected subthreshold (*p* = 7.28489e-08) signal at chromosome 6 in the superior nasal sector analyses, containing gene *EYS*. *EYS* is expressed in the retina and may play a role in the stability of the ciliary axoneme in both rods and cones (Alfano et al., 2016; McGuigan et al., 2017). Genetic variants in this gene are associated with retinitis pigmentosa (Messchaert et al., 2018; Yang et al., 2020; Suvannaboon et al., 2022). Genomic inflation factors range from 1.02 to 1.05, suggesting population structure was adequately controlled for. The lack of inflation in the tails of the Q-Q plots could be considered as evidence against a polygenic trait, although the dataset is of a limited size. Results are shown in Supplementary Figures S1–S9.

### 3.3. Gene-based testing

We next applied gene-based testing to test the hypothesis that predictors of ppRNFL thinning (as a marker of VAVFL) could be identified at the genic level rather than the univariate level. None of the 9 quantitative OCT measurement analyses reached the significance threshold *p* < 2.71× 10^−7^ (see methods).

We then compared the gene-based testing results with previously published gene-expression analysis. Walters et al. identified 26 ocular function genes, that showed evidence of differential expression in mice exposed to VGB, compared to the controls (Walters et al., 2020). We examined these 26 genes in our analyses to see if there was an enrichment of gene-based signal. Of the 26 candidate genes, we observed nominal significance for *SLC25A13*, *ENPP2*, and *CALCRL,* but none survived correction for multiple testing (see Table 2).

**Table 2:** Results of the VEGAS2 gene-based testing analysis, for *SLC25A13*, *ENPP2*, and *CALCRL.*

Analysis	*SLC25A13 P* value	*ENPP2 P* value	*CALCRL P* value
Average RNFL	NS	NS	NS
Nasal quad	0.026	NS	0.04
Inferior quad	NS	NS	NS
Superior quad	NS	0.016	NS
Temporal quad	NS	NS	NS
NI sector	0.017	0.041	0.016
IN sector	NS	NS	NS
SN sector	NS	NS	NS
NS sector	0.017	NS	NS

Analysis is the 9 different OCT measurement analysis, *SLC25A13 P* value, the *p* value for the *SLC25A13* gene; *ENPP2* *p* value, the *p* value for the *ENPP2* gene; *CALCRL p* value, *p* value for the CALCRL gene; NS, not significant.

### 3.4. Polygenic risk scoring

**Figure 2:**
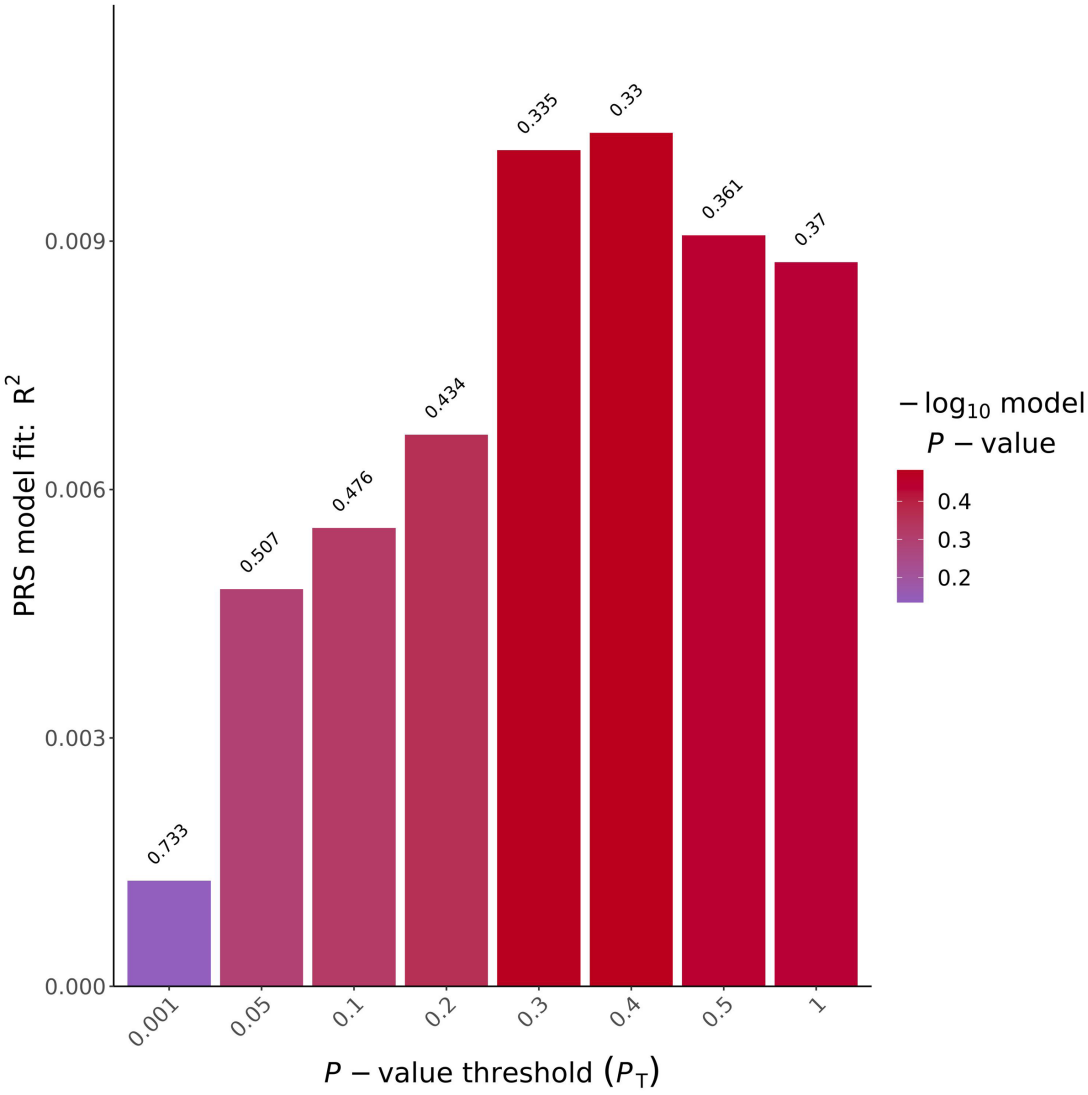
Average ppRNFL PRS results bar plot. *X*-axis shows the different *p* value thresholds used and the *y* axis shows the *R*^2^ of the PRS model used, on top of each bar plot is the uncorrected *p* value for that analysis.

To determine if individuals exposed to VGB had a predisposition to having a thinner RNFL as quantified by a PRS, we tested the correlation between polygenic burden for retinal thickness in our samples, to determine if our cohort were predisposed to having a thinner retina using previously published summary statistics for retinal thickness (Currant et al., 2021) (see methods). These analyses did not produce an association significant after correction for multiple testing. (See Table 3; Figure 2). for results of the average RNFL thickness PRS and Supplementary Tables S1–S8 and for the other 8 OCT measurements.

**Table 3:** Results for retinal thickness PRS correlated with average ppRNFL in VGB-exposed patients.

Threshold	*R* ^2^	*P*	Corrected *P*	Coefficient	Standard. Error	Num_SNP
0.001	0.0012736	0.732986	1	−293.999	858.39	1,138
0.05	0.00479505	0.507351	1	3337.01	5007.94	20,113
0.1	0.00553635	0.476091	1	4613.03	6439.14	32,740
0.2	0.00666373	0.434109	1	7225.86	9185.69	52,682
0.3	0.0100995	0.334856	1	10,822	11145.5	68,368
0.4	0.0103076	0.329883	1	12876.3	13124.6	81,383
0.5	0.00907194	0.360931	1	13269.8	14431.1	92,413
1	0.00874725	0.369736	1	16789.6	18599.2	123,553

Table of the Results for retinal thickness PRS correlated with average ppRNFL in VGB-exposed patients. Threshold, the value of *p* threshold used; *R*^2^ = variance *P*, *p* value; Corrected *P* value, *p* value corrected for the number of thresholds (*n* = 8) and number of OCT measurements tested (*n* = 9); coefficient, regression coefficient of the model; standard error, standard error; Num_SNP, number of SNPs included in the model.

### 3.5. Power calculation

Our power calculations (see methods) for the GWAS analysis indicated we were powered (80%) to detect a variant that explains 56% of the trait variance. Results are shown in the Supplementary Figure S10.

## 4. Discussion

We set out to identify common genetic predictors for VGB-induced ppRNFL thinning (as a marker of VAVFL). We did this under univariate, multivariate, and polygenic models with GWAS data and a range of OCT measurements. We were unable to identify a significant association with ppRNFL thinning in individuals exposed to VGB under any of the models tested.

However, when the VEGAS2 results were compared with previously published gene expression analysis, we found *SLC25A13* was nominally significant for 3 of the 9 OCT traits tested, *ENPP2* was found to be nominally significant in 2 out of 9 OCT traits tested and *CALCRL* was also found to be significant in 2 out of 9 OCT traits tested. Mutations in *SLC25A13* can cause citrin deficiency, which may result in neonatal intrahepatic cholestasis (Kobayashi et al., 1999; Nguyen et al., 2023). Visual dysfunction can occur in individuals with cholestasis (Fahnehjelm et al., 2011). *ENPP2* encodes autotaxin, which has phosphodiesterase and phospholipase activity (Koike et al., 2009; Perrakis and Moolenaar, 2014). Studies have shown that individuals with glaucoma have increased levels of autotaxin in their aqueous humor (Honjo et al., 2018; Ho et al., 2020). Cao et al., showed that SNPs that map to *CALCRL* are associated with actuate primary angle closure glaucoma (Cao et al., 2009). More work is needed to determine the potential link between dysregulation of *SCL25A13, ENPP2 and CALCRL* and ppRNFL thinning in individuals exposed to VGB.

Our PRS analysis set out to determine if individuals exposed to VGB had an increased polygenic burden of having a thinner retinal nerve fiber layer. These quantitative PRS analyses were all negative, with no association between ppRNFL thickness and exposure to VGB. A limitation of this analysis was that the cohort was not stratified by presence of VAVFL.

A major limitation of this study was the sample size. A larger sample size would obviously provide more power to identify a pharmacogenomic association, but recruitment is a challenge as VGB is rarely prescribed today in adults because of this adverse reaction. However, our results suggest that a common pharmacogenomic variant explaining >56% of risk of developing ppRNFL thinning caused by exposure to VGB is unlikely to exist. As well as the limited sample size, study participants were treated with VGB many years ago, and covariate information was sometimes missing. Our study participants were all of European ancestry, so further work is needed in other ethnic backgrounds.

A larger international consortium/effort to identify genetic predictors for ppRNFL thinning in individuals exposed to VGB, could enable a GWAS (or meta-analysis) with more participants, to increase statistical power. As this drug is still commonly prescribed in children with infantile spasms with TSCs, it would be also be possible to conduct a longitudinal genetic study to identify if these children also develop ppRNFL thinning due to exposure to VGB.

In conclusion, this study suggests that if pharmacogenomic predictors of VGB-induced ppRNFL thinning (as a marker of VAVFL) exist, they are likely to be of relatively small effect size or are driven by rare variants. Further analyses will need larger numbers or sequencing of rare variants.

## 5. Contribution to the field statement

Vigabatrin is an effective drug in the treatment of epilepsy. However, its use is limited by drug-associated permanent visual field loss. Identifying genetic predictors of this adverse reaction could enable safer, more widespread use of an otherwise very effective treatment for seizure control. In this context, we conducted various univariate and polygenic assessments of the role of common genetic variation, at the genomic level, in predicting this adverse drug reaction. We did not detect any effects that survived multiple correction. This work is an important contribution to the field as it suggests that common, univariate genetic predictors of clinically relevant effect (defined here as a variant explaining >56% of trait variance) probably do not exist for this adverse reaction. The work would suggest focusing genetic efforts on rare variants, detectable by exome and genome sequencing.

## Data availability statement

The data analyzed in this study is subject to the following licenses/restrictions: the raw SNP datasets presented in this article are not readily available due to ethical and privacy restrictions. The GWAS summary statistics data that support the findings of this study are available upon request. Requests to access these datasets should be directed to GC, gcavalleri@rcsi.ie.

## Ethics statement

The studies involving humans were approved by the Ethical approval was provided by the relevant ethics boards at each study site; Beaumont Hospital (study code 14/44). The University College London (UCL) Queen Square Institute of Neurology (study code 11/LO/2016) and the University Medical Centre, Utrecht (study codes 09/352 and 18–466). The studies were conducted in accordance with the local legislation and institutional requirements. Written informed consent for participation in this study was provided by the participants’ legal guardians/next of kin.

## Author contributions

All authors listed have made a substantial, direct, and intellectual contribution to the work and approved it for publication.

## Group members of EpiPGX Consortium

Joseph Willis, University College London, London, UK; Mojgansadat Borghei, Université Libre de Bruxelles, Brussels, Belgium; Simona Donatello, Université Libre de Bruxelles, Brussels, Belgium; Martin J. Brodie, University of Glasgow, Glasgow, UK; Pauls Auce, St George’s University Hospitals NHS Foundation Trust; Andrea Jorgensen, University of Liverpool, Liverpool, UK; Sarah R. Langley, Imperial College London, London, UK; Yvonne Weber, University of Tübingen, Tübingen, Germany; Christian Hengsbach, University of Tübingen, Tübingen, Germany; Martin Krenn, University of Vienna, Vienna, Austria; Fritz Zimprich, University of Vienna, Vienna, Austria; Ekaterina Pataraia, University of Vienna, Vienna, Austria; Karl Martin Klein, Philipps-Universität Marburg, Marburg, Germany; Hiltrud Muhle, Universitätsklinikum Schleswig - Holstein, Kiel, Germany; Rikke S. Møller, Danish Epilepsy Centre, Dianalund, Denmark; Marina Nikanorova, Danish Epilepsy Centre, Dianalund, Denmark; Stefan Wolking, Neurology and Epileptology, Hertie Institute for Clinical Brain Research, University of Tübingen, Tübingen, Germany; Ellen Campbell, Belfast Health and Social Care Trust. Belfast, United Kingdom; Antonella Riva, University of Genova, Genova, Italy; Marcello Scala, University of Genova, Genova, Italy.

## Funding

This work was supported by a grant from the European Commission (7th Framework Programme Grant 279062, EpiPGX) and in part by a research grant from Science Foundation Ireland (SFI) under grant numbers 16/RC/3948 and co-funded under the European Regional Development Fund and by FutureNeuro industry partners and by Science Foundation Ireland through the SFI Centre for Research Training in Genomics Data Science under grant number 18/CRT/6214. Research supported by PNRR-MUR-M4C2 PE0000006 Research Program “MNESYS”—A multiscale integrated approach to the study of the nervous system in health and disease. IRCCS ‘G. Gaslini’ is a member of ERN-Epicare. This work was also supported by the Epilepsy Society, United Kingdom.

## Conflict of interest

The authors declare that the research was conducted in the absence of any commercial or financial relationships that could be construed as a potential conflict of interest.

## Publisher’s note

All claims expressed in this article are solely those of the authors and do not necessarily represent those of their affiliated organizations, or those of the publisher, the editors and the reviewers. Any product that may be evaluated in this article, or claim that may be made by its manufacturer, is not guaranteed or endorsed by the publisher.

## References

[ref1] Alfano G. Kruczek P. M. Shah A. Z. Kramarz B. Jeffery G. Zelhof A. C.. (2016). EYS is a protein associated with the ciliary Axoneme in rods and cones. PLoS One 11:e0166397. doi: 10.1371/journal.pone.0166397, PMID: 27846257 PMC5112921

[ref2] Ben-Menachem E. (2011). Mechanism of action of vigabatrin: correcting misperceptions. Acta Neurol. Scand. Suppl. 124, 5–15. doi: 10.1111/j.1600-0404.2011.01596.x, PMID: 22061176

[ref3] Biswas A. Yossofzai O. Vincent A. Go C. Widjaja E. (2020). Vigabatrin-related adverse events for the treatment of epileptic spasms: systematic review and meta-analysis. Expert. Rev. Neurother. 20, 1315–1324. doi: 10.1080/14737175.2020.1840356, PMID: 33078964

[ref4] Bresnahan R. Gianatsi M. Maguire M. J. Tudur Smith C. Marson A. G. (2020). Vigabatrin add-on therapy for drug-resistant focal epilepsy. Cochrane Database Syst. Rev. 7:CD007302. doi: 10.1002/14651858.CD007302.pub3 32730657 PMC8211760

[ref5] Cao D. Liu X. Guo X. Cong Y. Huang J. Mao Z. (2009). Investigation of the association between CALCRL polymorphisms and primary angle closure glaucoma. Mol. Vis. 15, 2202–2208. PMID: 19898635 PMC2773738

[ref6] Chan K. Hoon M. Pattnaik B. R. Ver Hoeve J. N. Wahlgren B. Gloe S.. (2020). Vigabatrin-induced retinal functional alterations and second-order neuron plasticity in C57BL/6J mice. Invest. Ophthalmol. Vis. Sci. 61:17. doi: 10.1167/iovs.61.2.17, PMID: 32053727 PMC7326505

[ref7] Chiron C. (2016). Stiripentol and vigabatrin current roles in the treatment of epilepsy. Expert. Opin. Pharmacother. 17, 1091–1101. doi: 10.1517/14656566.2016.1161026, PMID: 26933940

[ref8] Chiron C. Dumas C. Jambaqué I. Mumford J. Dulac O. (1997). Randomized trial comparing vigabatrin and hydrocortisone in infantile spasms due to tuberous sclerosis. Epilepsy Res. 26, 389–395. doi: 10.1016/S0920-1211(96)01006-6, PMID: 9095401

[ref9] Choi S. W. O'reilly P. F. (2019). PRSice-2: polygenic risk score software for biobank-scale data. Gigascience 8:giz082. doi: 10.1093/gigascience/giz082, PMID: 31307061 PMC6629542

[ref10] Clayton L. M. Devile M. Punte T. De Haan G. J. Sander J. W. Acheson J. F.. (2012). Patterns of peripapillary retinal nerve fiber layer thinning in vigabatrin-exposed individuals. Ophthalmology 119, 2152–2160. doi: 10.1016/j.ophtha.2012.05.009, PMID: 22853973

[ref11] Clayton L. M. Dévilé M. Punte T. Kallis C. De Haan G. J. Sander J. W.. (2011). Retinal nerve fiber layer thickness in vigabatrin-exposed patients. Ann. Neurol. 69, 845–854. doi: 10.1002/ana.22266, PMID: 21246602

[ref12] Clayton L. M. Stern W. M. Newman W. D. Sander J. W. Acheson J. Sisodiya S. M. (2013). Evolution of visual field loss over ten years in individuals taking vigabatrin. Epilepsy Res. 105, 262–271. doi: 10.1016/j.eplepsyres.2013.02.014, PMID: 23541931

[ref13] Currant H. Hysi P. Fitzgerald T. W. Gharahkhani P. Bonnemaijer P. W. M. Senabouth A.. (2021). Genetic variation affects morphological retinal phenotypes extracted from UK biobank optical coherence tomography images. PLoS Genet. 17:e1009497. doi: 10.1371/journal.pgen.1009497, PMID: 33979322 PMC8143408

[ref14] Davies J. A. (1995). Mechanisms of action of antiepileptic drugs. Seizure 4, 267–271. doi: 10.1016/S1059-1311(95)80003-4 8719918

[ref15] Duboc A. Hanoteau N. Simonutti M. Rudolf G. Nehlig A. Sahel J. A.. (2004). Vigabatrin, the GABA-transaminase inhibitor, damages cone photoreceptors in rats. Ann. Neurol. 55, 695–705. doi: 10.1002/ana.20081, PMID: 15122710

[ref16] Eke T. Talbot J. F. Lawden M. C. (1997). Severe persistent visual field constriction associated with vigabatrin. BMJ 314, 180–181. doi: 10.1136/bmj.314.7075.180, PMID: 9022432 PMC2125673

[ref17] EMA (2018). Kigabeq (vigabatrin) an overview of Kigabeq and why it is authorised in the EU. Available at: https://www.ema.europa.eu/en/documents/overview/kigabeq-epar-medicine-overview_en.pdf

[ref18] Fahnehjelm K. T. Fischler B. Martin L. Nemeth A. (2011). Occurrence and pattern of ocular disease in children with cholestatic disorders. Acta Ophthalmol. 89, 143–150. doi: 10.1111/j.1755-3768.2009.01671.x, PMID: 20384607

[ref19] FDA (2020). Sabril, full prescribing information. Available at: https://www.accessdata.fda.gov/drugsatfda_docs/label/2020/020427s021,022006s023lbl.pdf

[ref20] Hancock E. Osborne J. P. (1999). Vigabatrin in the treatment of infantile spasms in tuberous sclerosis: literature review. J. Child Neurol. 14, 71–74. doi: 10.1177/088307389901400201, PMID: 10073425

[ref21] Heim M. K. Gidal B. E. (2012). Vigabatrin-associated retinal damage – potential biochemical mechanisms. Acta Neurol. Scand. 126, 219–228. doi: 10.1111/j.1600-0404.2012.01684.x, PMID: 22632110

[ref22] Hisama F. M. Mattson R. H. Lee H. H. Felice K. Petroff O. A. C. (2001). GABA and the ornithineδ-aminotransferase gene in vigabatrin-associated visual field defects. Seizure 10, 505–507. doi: 10.1053/seiz.2001.0524, PMID: 11749107

[ref23] Ho L. T. Y. Osterwald A. Ruf I. Hunziker D. Mattei P. Challa P.. (2020). Role of the autotaxin-lysophosphatidic acid axis in glaucoma, aqueous humor drainage and fibrogenic activity. Biochim. Biophys. Acta Mol. Basis Dis. 1866:165560. doi: 10.1016/j.bbadis.2019.165560, PMID: 31648019 PMC6863611

[ref24] Honjo M. Igarashi N. Kurano M. Yatomi Y. Igarashi K. Kano K.. (2018). Autotaxin-lysophosphatidic acid pathway in intraocular pressure regulation and Glaucoma subtypes. Invest. Ophthalmol. Vis. Sci. 59, 693–701. doi: 10.1167/iovs.17-23218, PMID: 29392315

[ref25] Jacob J. N. Hesse G. W. Shashoua V. E. (1990). Synthesis, brain uptake, and pharmacological properties of a glyceryl lipid containing GABA and the GABA-T inhibitor gamma-vinyl-GABA. J. Med. Chem. 33, 733–736. doi: 10.1021/jm00164a042, PMID: 2299639

[ref26] Jammoul F. Dégardin J. Pain D. Gondouin P. Simonutti M. Dubus E.. (2010). Taurine deficiency damages photoreceptors and retinal ganglion cells in vigabatrin-treated neonatal rats. Mol. Cell. Neurosci. 43, 414–421. doi: 10.1016/j.mcn.2010.01.008, PMID: 20132888 PMC2864319

[ref27] Kinirons P. Cavalleri G. L. Singh R. Shahwan A. Acheson J. F. Wood N. W.. (2006). A pharmacogenetic exploration of vigabatrin-induced visual field constriction. Epilepsy Res. 70, 144–152. doi: 10.1016/j.eplepsyres.2006.03.012, PMID: 16675198

[ref28] Kjellström U. Andréasson S. Ponjavic V. (2014). Attenuation of the retinal nerve fibre layer and reduced retinal function assessed by optical coherence tomography and full-field electroretinography in patients exposed to vigabatrin medication. Acta Ophthalmol. 92, 149–157. doi: 10.1111/aos.12030, PMID: 23387307

[ref29] Kobayashi K. Sinasac D. S. Iijima M. Boright A. P. Begum L. Lee J. R.. (1999). The gene mutated in adult-onset type II citrullinaemia encodes a putative mitochondrial carrier protein. Nat. Genet. 22, 159–163. doi: 10.1038/9667, PMID: 10369257

[ref30] Koike S. Keino-Masu K. Ohto T. Sugiyama F. Takahashi S. Masu M. (2009). Autotaxin/Lysophospholipase D-mediated lysophosphatidic acid signaling is required to form distinctive large lysosomes in the visceral endoderm cells of the mouse yolk sac*. J. Biol. Chem. 284, 33561–33570. doi: 10.1074/jbc.M109.012716, PMID: 19808661 PMC2785199

[ref31] Lawden M. C. Eke T. Degg C. Harding G. F. Wild J. M. (1999). Visual field defects associated with vigabatrin therapy. J. Neurol. Neurosurg. Psychiatry 67, 716–722. doi: 10.1136/jnnp.67.6.716, PMID: 10567485 PMC1736662

[ref32] Lawthom C. Smith P. E. M. Wild J. M. (2009). Nasal retinal nerve Fiber layer attenuation: a biomarker for Vigabatrin toxicity. Ophthalmology 116, 565–571. doi: 10.1016/j.ophtha.2008.09.047, PMID: 19168223

[ref33] Liu J. Z. Mcrae A. F. Nyholt D. R. Medland S. E. Wray N. R. Brown K. M.. (2010). A versatile gene-based test for genome-wide association studies. Am. J. Hum. Genet. 87, 139–145. doi: 10.1016/j.ajhg.2010.06.009, PMID: 20598278 PMC2896770

[ref34] Maguire M. J. A. H. K. A. W. J. M. A. H. J. L. A. M. A. G. (2010). Prevalence of visual field loss following exposure to vigabatrin therapy: a systematic review. Epilepsia 51, 2423–2431. doi: 10.1111/j.1528-1167.2010.02772.x, PMID: 21070215

[ref35] Marchini J. Howie B. Myers S. Mcvean G. Donnelly P. (2007). A new multipoint method for genome-wide association studies by imputation of genotypes. Nat. Genet. 39, 906–913. doi: 10.1038/ng2088, PMID: 17572673

[ref36] Marino R. Jr. Rasmussen T. (1968). Visual field changes after temporal lobectomy in man. Neurology 18, 825–835. doi: 10.1212/WNL.18.9.825, PMID: 5693676

[ref37] Mccormack M. Gui H. Ingason A. Speed D. Wright G. E. B. Zhang E. J.. (2018). Genetic variation in CFH predicts phenytoin-induced maculopapular exanthema in European-descent patients. Neurology 90, e332–e341. doi: 10.1212/WNL.0000000000004853, PMID: 29288229 PMC5798660

[ref38] Mcguigan D. B. Heon E. Cideciyan A. V. Ratnapriya R. Lu M. Sumaroka A.. (2017). EYS mutations causing autosomal recessive retinitis Pigmentosa: changes of retinal structure and function with disease progression. Genes 8:178. doi: 10.3390/genes8070178 28704921 PMC5541311

[ref39] Messchaert M. Haer-Wigman L. Khan M. I. Cremers F. P. M. Collin R. W. J. (2018). EYS mutation update: in silico assessment of 271 reported and 26 novel variants in patients with retinitis pigmentosa. Hum. Mutat. 39, 177–186. doi: 10.1002/humu.23371, PMID: 29159838

[ref40] Messer R. Knupp K. G. (2020). Infantile spasms: opportunities to improve care. Semin. Neurol. 40, 236–245. doi: 10.1055/s-0040-1705121, PMID: 32143232

[ref41] Mishra A. Macgregor S. (2015). VEGAS2: software for more flexible gene-based testing. Twin Res. Hum. Genet. 18, 86–91. doi: 10.1017/thg.2014.79, PMID: 25518859

[ref42] Moseng L. Sæter M. Mørch-Johnsen G. H. Hoff J. M. Gajda A. Brodtkorb E.. (2011). Retinal nerve fibre layer attenuation: clinical indicator for vigabatrin toxicity. Acta Ophthalmol. 89, 452–458. doi: 10.1111/j.1755-3768.2010.02077.x, PMID: 21251242

[ref43] Nguyen M.-H. T. Nguyen A.-H. P. Ngo D.-N. Nguyen P.-M. T. Tang H.-S. Giang H.. (2023). The mutation spectrum of SLC25A13 gene in citrin deficiency: identification of novel mutations in Vietnamese pediatric cohort with neonatal intrahepatic cholestasis. J. Hum. Genet. 68, 305–312. doi: 10.1038/s10038-022-01112-2, PMID: 36599957

[ref44] Nousiainen I. Mantyjarvi M. Kalviainen R. (2001). No reversion in vigabatrin-associated visual field defects. Neurology 57, 1916–1917. doi: 10.1212/WNL.57.10.1916, PMID: 11723291

[ref45] Paul S. R. Krauss G. L. Miller N. R. Medura M. T. Miller T. A. Johnson M. A. (2001). Visual function is stable in patients who continue long-term vigabatrin therapy: implications for clinical decision making. Epilepsia 42, 525–530. doi: 10.1046/j.1528-1157.2001.49299.x, PMID: 11440348

[ref46] Perrakis A. Moolenaar W. H. (2014). Autotaxin: structure-function and signaling. J. Lipid Res. 55, 1010–1018. doi: 10.1194/jlr.R046391, PMID: 24548887 PMC4031933

[ref47] Purcell S. Neale B. Todd-Brown K. Thomas L. Ferreira M. A. Bender D.. (2007). PLINK: a tool set for whole-genome association and population-based linkage analyses. Am. J. Hum. Genet. 81, 559–575. doi: 10.1086/519795, PMID: 17701901 PMC1950838

[ref48] R Core Team (2022). R: A language and Environment for statistical computing R foundation for statistical computing. Vienna, Austria: R Foundation for Statistical Computing. Available at: https://www.R-project.org

[ref49] Russell-Eggitt I. M. Mackey D. A. Taylor D. S. I. Timms C. Walker J. W. (2000). Vigabatrin-associated visual field defects in children. Eye 14, 334–339. doi: 10.1038/eye.2000.83, PMID: 11026995

[ref50] Schubert-Bast S. Strzelczyk A. (2021). Review of the treatment options for epilepsy in tuberous sclerosis complex: towards precision medicine. Ther. Adv. Neurol. Disord. 14:17562864211031100. doi: 10.1177/17562864211031100 34349839 PMC8290505

[ref51] Suvannaboon R. Pawestri A. R. Jinda W. Tuekprakhon A. Trinavarat A. Atchaneeyasakul L.-O. (2022). Genotypic and phenotypic profiles of EYS gene-related retinitis pigmentosa: a retrospective study. Sci. Rep. 12:21494. doi: 10.1038/s41598-022-26017-0, PMID: 36513702 PMC9748023

[ref52] Turner S. (2018). Qqman: an R package for visualizing GWAS results using Q-Q and Manhattan plots. J. Open Source Softw. 3:731. doi: 10.21105/joss.00731

[ref53] Van Lanen R. Hoeberigs M. C. Bauer N. J. C. Haeren R. H. L. Hoogland G. Colon A.. (2018). Visual field deficits after epilepsy surgery: a new quantitative scoring method. Acta Neurochir. 160, 1325–1336. doi: 10.1007/s00701-018-3525-9, PMID: 29623432 PMC5995984

[ref54] Vigevano F. Cilio M. R. (1997). Vigabatrin versus ACTH as first-line treatment for infantile spasms: a randomized, prospective study. Epilepsia 38, 1270–1274. doi: 10.1111/j.1528-1157.1997.tb00063.x 9578521

[ref55] Vogel K. R. Ainslie G. R. Schmidt M. A. Wisor J. P. Gibson K. M. (2017). mTOR inhibition mitigates molecular and biochemical alterations of Vigabatrin-induced visual field toxicity in mice. Pediatr. Neurol. 66, 44–52.e1. doi: 10.1016/j.pediatrneurol.2016.09.016, PMID: 27816307 PMC5866057

[ref56] Walters D. Vogel K. R. Brown M. Shi X. Roullet J. B. Gibson K. M. (2020). Transcriptome analysis in mice treated with vigabatrin identifies dysregulation of genes associated with retinal signaling circuitry. Epilepsy Res. 166:106395. doi: 10.1016/j.eplepsyres.2020.106395, PMID: 32679486 PMC7494645

[ref57] Wang Q. P. Jammoul F. Duboc A. Gong J. Simonutti M. Dubus E.. (2008). Treatment of epilepsy: the GABA-transaminase inhibitor, vigabatrin, induces neuronal plasticity in the mouse retina. Eur. J. Neurosci. 27, 2177–2187. doi: 10.1111/j.1460-9568.2008.06175.x, PMID: 18412635 PMC2933832

[ref58] Wickham H. (2016). G gplot2: Elegant graphics for data analysis, Springer-Verlag New York.

[ref59] Wild J. M. Martinez C. Reinshagen G. Harding G. F. (1999). Characteristics of a unique visual field defect attributed to vigabatrin. Epilepsia 40, 1784–1794. doi: 10.1111/j.1528-1157.1999.tb01599.x, PMID: 10612345

[ref60] Yang L. Fujinami K. Ueno S. Kuniyoshi K. Hayashi T. Kondo M.. (2020). Genetic Spectrum of EYS-associated retinal disease in a large Japanese cohort: identification of disease-associated variants with relatively high allele frequency. Sci. Rep. 10:5497. doi: 10.1038/s41598-020-62119-3, PMID: 32218477 PMC7099090

[ref61] Yee J. M. Agulian S. Kocsis J. D. (1998). Vigabatrin enhances promoted release of GABA in neonatal rat optic nerve. Epilepsy Res. 29, 195–200. doi: 10.1016/S0920-1211(97)00086-7, PMID: 9551781

