# Supplementary material for: Testing for pharmacogenomic predictors of ppRNFL thinning in individuals exposed to vigabatrin
Source: Front Neurosci. 2023 Sep 8;17:1156362. doi: 10.3389/fnins.2023.1156362 (PMC10542409; doi:10.3389/fnins.2023.1156362)
Supplement: Supplementary file 1 [file Data_Sheet_1.docx]

**Supplementary figures**

**Quantitative GWAS Manhattan plots and QQ plots**

Average ppRNFL OCT GWAS


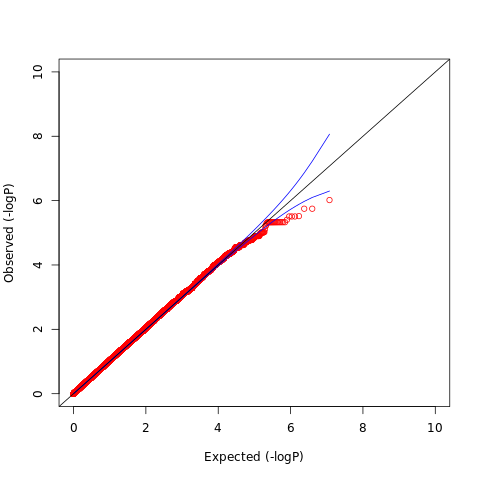


B

A


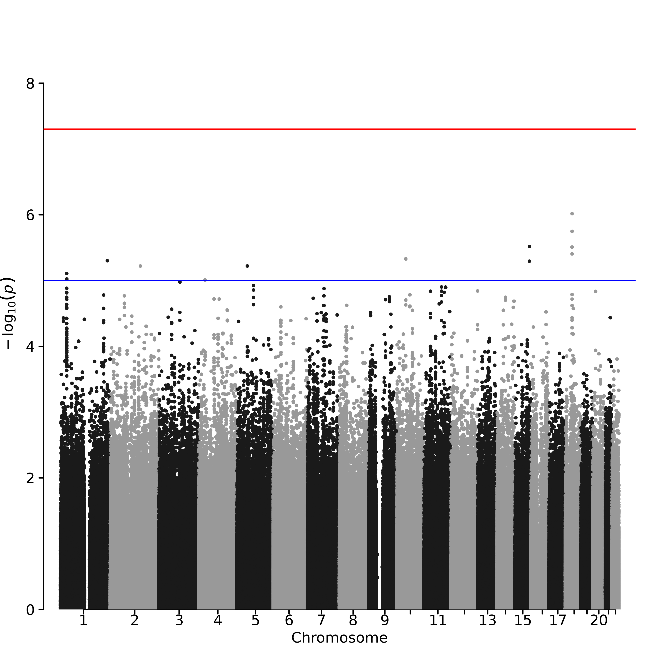


Figure 1. A) Manhattan plot of the average ppRNFL OCT measurement GWAS. This analysis was negative. B) QQ plot of the average ppRNFL OCT measurement GWAS. GIF = 1.04.

Inferior quadrant OCT GWAS


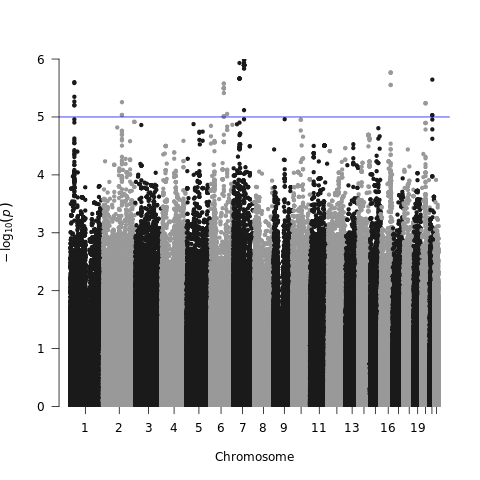


A

B


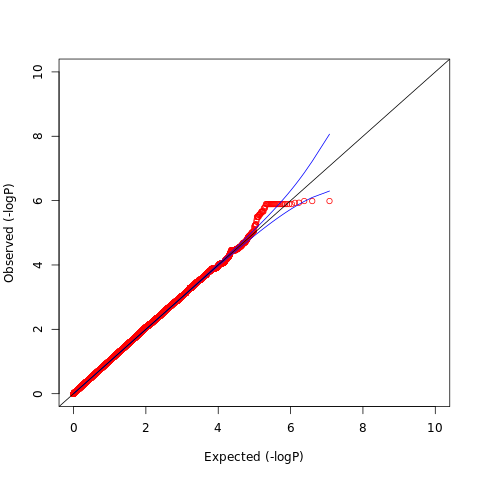
Figure 2. A) Manhattan plot of the inferior quadrant OCT measurement GWAS. This analysis was negative. B) QQ plot of the inferior quadrant OCT measurement GWAS. GIF = 1.04.

Nasal quadrant OCT GWAS

B

A


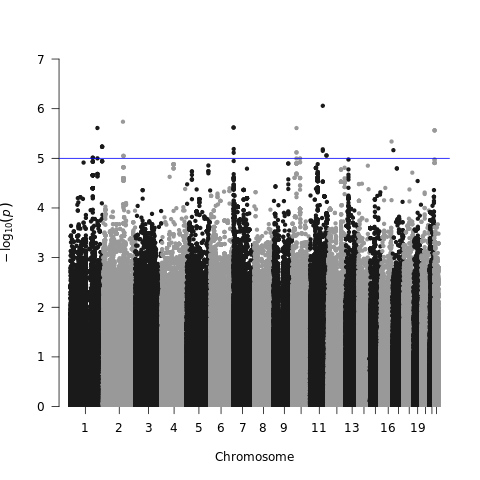

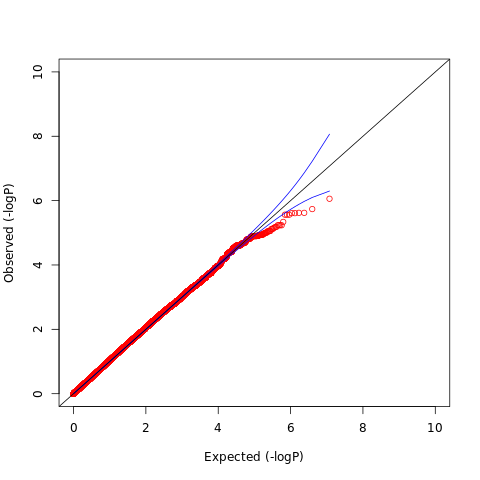


Figure 3. A) Manhattan plot of the nasal quadrant OCT measurement GWAS. This analysis was negative. B) QQ plot of the nasal quadrant OCT measurement GWAS. The GIF =1.03.

Superior quadrant OCT GWAS

B

A


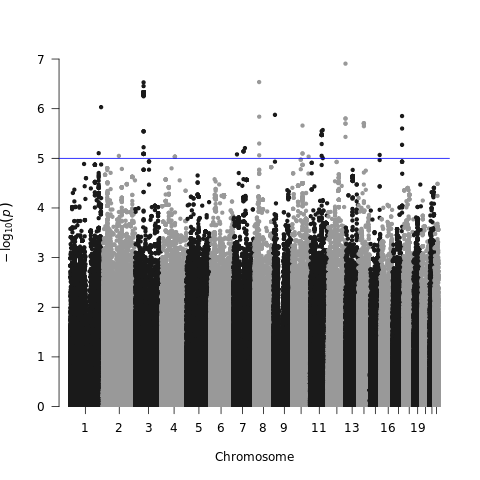


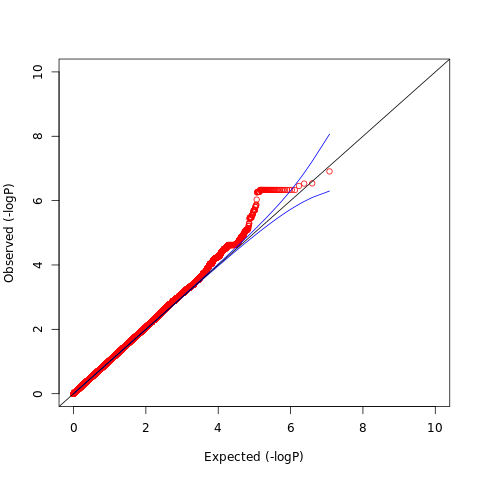


Figure 4. A) Manhattan plot of the superior quadrant OCT measurement GWAS. This analysis was negative. B) QQ plot of the superior quadrant OCT measurement GWAS. GIF = 1.05.

Temporal quadrant OCT GWAS

A

B


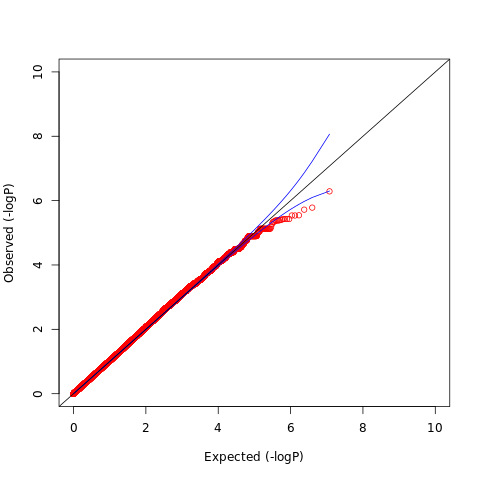


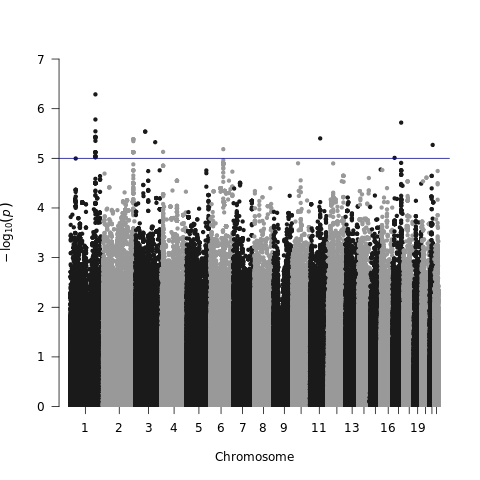


Figure 5. A) Manhattan plot of the temporal quadrant OCT measurement GWAS. This analysis was negative. B) QQ plot of the temporal quadrant OCT measurement GWAS. GIF = 1.03.

Inferior nasal sector OCT GWAS

B

A


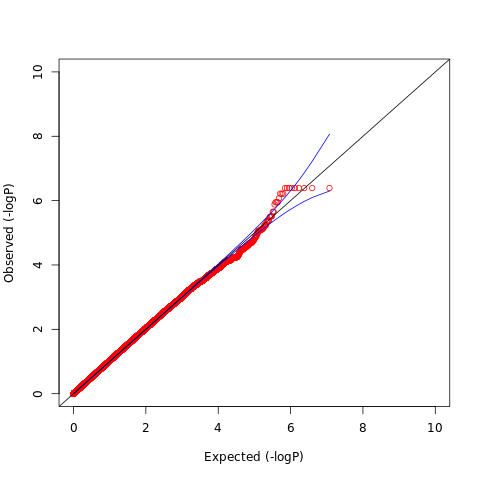

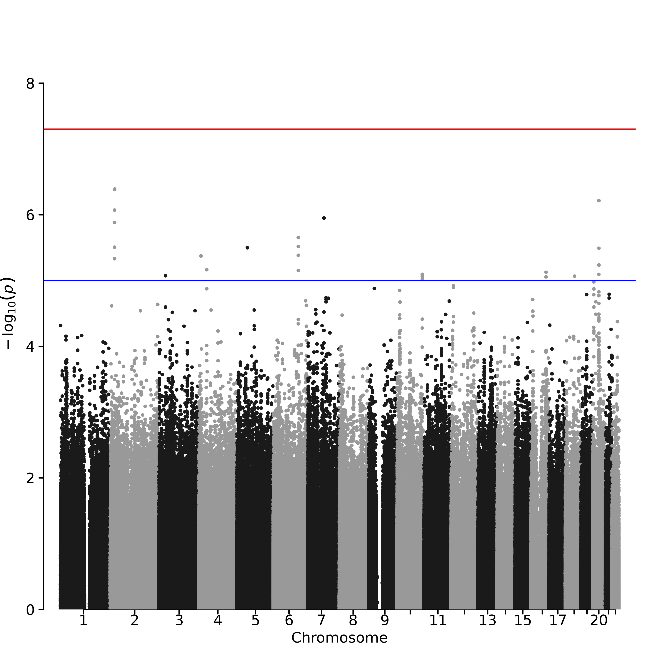


Figure 6. A) Manhattan plot of the inferior nasal sector OCT measurement GWAS. This analysis was negative. B) QQ plot of the inferior nasal sector OCT measurement GWAS. GIF = 1.03.

Superior nasal sector OCT GWAS

B

A


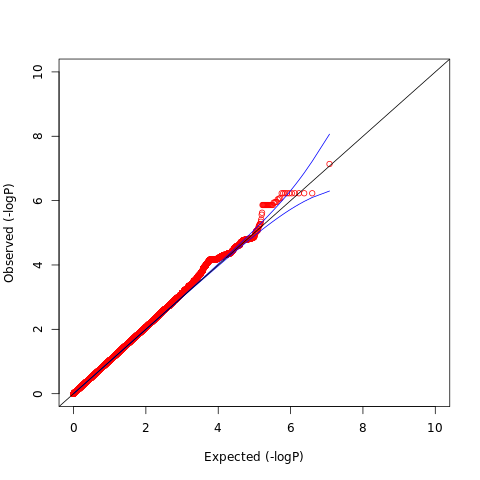

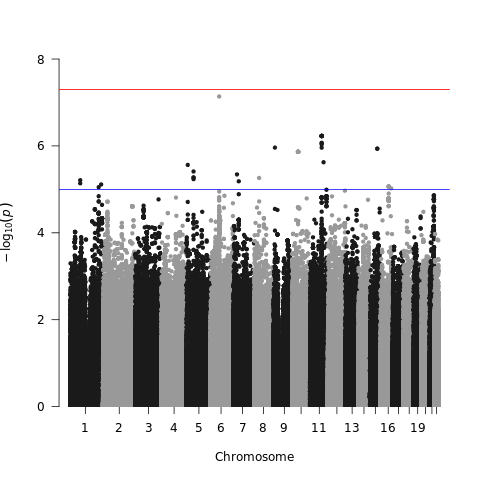


Figure 7. A) Manhattan plot of the superior nasal sector OCT measurement GWAS. This analysis was negative. B) QQ plot of the superior nasal sector OCT measurement GWAS. GIF = 1.04

Nasal superior sector OCT GWAS

B

A


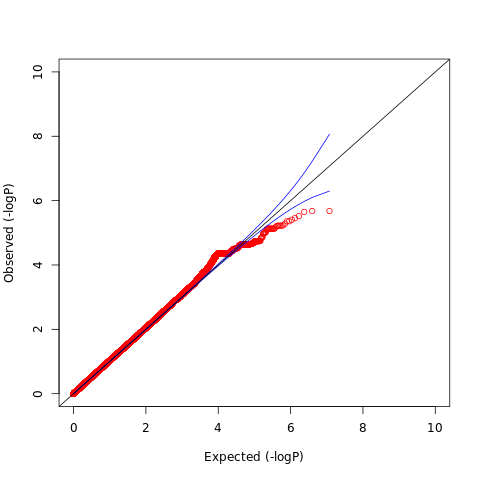


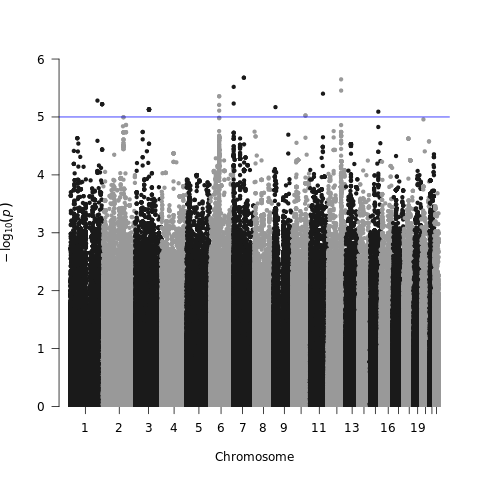


Figure 8. A) Manhattan plot of the nasal superior sector OCT measurement GWAS. This analysis was negative. B) QQ plot of the nasal superior sector OCT measurement GWAS. GIF =1.04.

Nasal inferior sector OCT GWAS Manhattan plot

A

B


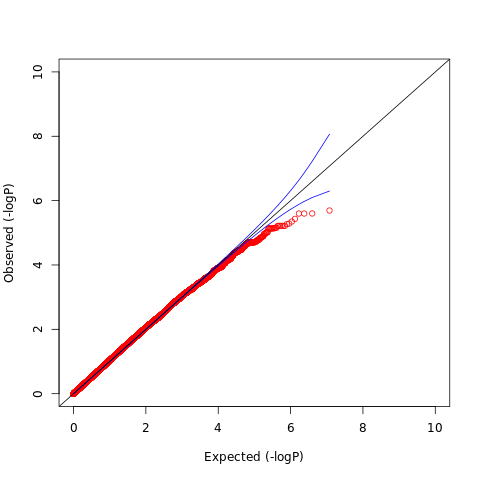


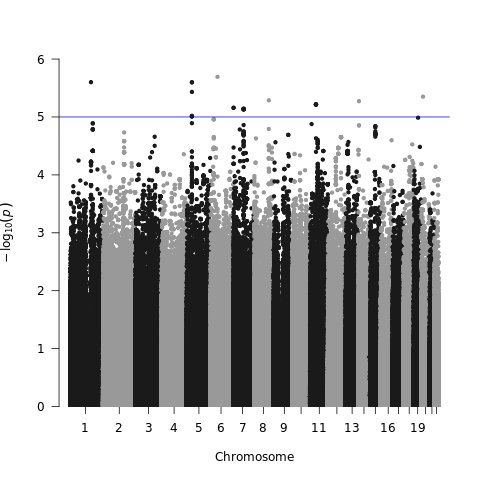


Figure 9. A) Manhattan plot of the nasal inferior sector OCT measurement GWAS. This analysis was negative. B) QQ plot of the nasal inferior sector OCT measurement GWAS. GIF = 1.04.

**PRS analysis**

Inferior quadrant PRS

| Threshold | R2 | P | Corrected P | Coefficient | Standard Error | No. of SNP |
| --- | --- | --- | --- | --- | --- | --- |
| 0.001 | 0.00295052 | 0.605039 | 1 | -798.97 | 1538.01 | 1138 |
| 0.05 | 0.00317828 | 0.5914 | 1 | 4850.75 | 8995.32 | 20113 |
| 0.1 | 0.00739166 | 0.412146 | 1 | 9516.94 | 11536 | 32740 |
| 0.2 | 0.0155266 | 0.232942 | 1 | 19693.4 | 16369.2 | 52682 |
| 0.3 | 0.0153124 | 0.236236 | 1 | 23792 | 19917 | 68368 |
| 0.4 | 0.0163402 | 0.220914 | 1 | 28946.4 | 23439 | 81383 |
| 0.5 | 0.0165069 | 0.218541 | 1 | 31959.5 | 25744.6 | 92413 |
| 1 | 0.0178087 | 0.20099 | 1 | 42773.2 | 33139.2 | 123553 |

Table 1. PRS analysis for the inferior quadrant PRS for retinal thickness. This analysis was negative. Threshold = the p-value threshold used, R2 = variance P = p value, Corrected P value = P value corrected for the number of thresholds (n =8) and number of OCT measurements tested (n = 9), coefficient = regression coefficient of the model, standard error = standard error, No. of SNP = number of SNPs included in the model.

Nasal quadrant PRS

| Threshold | R2 | P | Corrected P | Coefficient | Standard Error | No. of SNP |
| --- | --- | --- | --- | --- | --- | --- |
| 0.001 | 0.0110706 | 0.36741 | 1 | 715.065 | 788.277 | 1138 |
| 0.05 | 0.0187281 | 0.239813 | 1 | 5440.49 | 4589.6 | 20113 |
| 0.1 | 0.00970703 | 0.39889 | 1 | 5039.05 | 5937.24 | 32740 |
| 0.2 | 0.0100125 | 0.391518 | 1 | 7306.91 | 8475.4 | 52682 |
| 0.3 | 0.0111647 | 0.365366 | 1 | 9386.68 | 10303.4 | 68368 |
| 0.4 | 0.0096357 | 0.40064 | 1 | 10270.4 | 12146.2 | 81383 |
| 0.5 | 0.00798601 | 0.444439 | 1 | 10271 | 13356.1 | 92413 |
| 1 | 0.00478009 | 0.554532 | 1 | 10238.9 | 17243 | 123553 |

Table 2. PRS analysis for the nasal quadrant PRS for retinal thickness. See Table 1. for column titles explanations.

Superior quadrant PRS

| Threshold | R2 | P | Corrected P | Coefficient | Standard Error | No. of SNP |
| --- | --- | --- | --- | --- | --- | --- |
| 0.001 | 0.00123297 | 0.741731 | 1 | -450.422 | 1361.4 | 1138 |
| 0.05 | 0.0116539 | 0.308858 | 1 | 8100.47 | 7903.15 | 20113 |
| 0.1 | 0.011487 | 0.31238 | 1 | 10346.5 | 10168.7 | 32740 |
| 0.2 | 0.01358 | 0.271597 | 1 | 16061.8 | 14496.1 | 52682 |
| 0.3 | 0.0186381 | 0.1968 | 1 | 22891.5 | 17568.9 | 68368 |
| 0.4 | 0.0179628 | 0.205241 | 1 | 26467.6 | 20702.3 | 81383 |
| 0.5 | 0.0141873 | 0.261015 | 1 | 25839.2 | 22805.5 | 92413 |
| 1 | 0.0135546 | 0.272051 | 1 | 32543.3 | 29399 | 123553 |

Table 3. PRS analysis for the superior quadrant PRS for retinal thickness. See Table 1. for column titles explanations.

Temporal quadrant PRS

| Threshold | R2 | P | Corrected P | Coefficient | Standard Error | No. of SNP |
| --- | --- | --- | --- | --- | --- | --- |
| 0.001 | 0.00701161 | 0.488925 | 1 | -544.509 | 782.744 | 1138 |
| 0.05 | 0.0100163 | 0.40772 | 1 | -3806.98 | 4571.01 | 20113 |
| 0.1 | 0.0112524 | 0.380018 | 1 | -5191.14 | 5876.53 | 32740 |
| 0.2 | 0.0338246 | 0.125203 | 1 | -12850.3 | 8281.93 | 52682 |
| 0.3 | 0.0192645 | 0.249472 | 1 | -11797.9 | 10160.5 | 68368 |
| 0.4 | 0.0174345 | 0.273553 | 1 | -13218.6 | 11979.2 | 81383 |
| 0.5 | 0.0154086 | 0.303639 | 1 | -13651 | 13174.5 | 92413 |
| 1 | 0.0147189 | 0.314826 | 1 | -17191.3 | 16982.2 | 123553 |

Table 4. PRS analysis for the temporal quadrant PRS for retinal thickness. See Table 1. for column titles explanations.

Inferior nasal sector PRS

| Threshold | R2 | P | Corrected P | Coefficient | Standard Error | No. of SNPs |
| --- | --- | --- | --- | --- | --- | --- |
| 0.001 | 0.00215183 | 0.671517 | 1 | 674.007 | 1582.8 | 1138 |
| 0.05 | 0.0238917 | 0.154058 | 1 | 13137.6 | 9118.74 | 20113 |
| 0.1 | 0.0171714 | 0.228164 | 1 | 14328.7 | 11787.4 | 32740 |
| 0.2 | 0.0242482 | 0.150962 | 1 | 24310.9 | 16745.4 | 52682 |
| 0.3 | 0.0232121 | 0.160159 | 1 | 28936.5 | 20386.5 | 68368 |
| 0.4 | 0.0240512 | 0.152664 | 1 | 34690.7 | 23995.9 | 81383 |
| 0.5 | 0.0242584 | 0.150874 | 1 | 38271.7 | 26355.8 | 92413 |
| 1 | 0.0241295 | 0.151985 | 1 | 49182.3 | 33962.9 | 123553 |

Table 5. PRS analysis for the inferior nasal sector PRS for retinal thickness. See Table 1. for column titles explanations.

Superior nasal sector PRS

| Threshold | R2 | P | Corrected P | Coefficient | Standard Error | No. of SNP |
| --- | --- | --- | --- | --- | --- | --- |
| 0.001 | 4.60E-05 | 0.951288 | 1 | -90.2348 | 1471.89 | 1138 |
| 0.05 | 0.0224455 | 0.173405 | 1 | 11661.3 | 8479.92 | 20113 |
| 0.1 | 0.0178327 | 0.225805 | 1 | 13372.2 | 10944.2 | 32740 |
| 0.2 | 0.0257385 | 0.144379 | 1 | 22937.3 | 15540.7 | 52682 |
| 0.3 | 0.029822 | 0.115548 | 1 | 30036.3 | 18852.3 | 68368 |
| 0.4 | 0.0267293 | 0.136728 | 1 | 33490.9 | 22251.3 | 81383 |
| 0.5 | 0.0186266 | 0.215609 | 1 | 30711.6 | 24580.4 | 92413 |
| 1 | 0.0167456 | 0.2407 | 1 | 37521 | 31713 | 123553 |

Table 6. PRS analysis for the superior nasal sector PRS for retinal thickness. See Table 1. for column titles explanations.

Nasal superior sector PRS

| Threshold | R2 | P | Corrected P | Coefficient | Standard Error | No. of SNP |
| --- | --- | --- | --- | --- | --- | --- |
| 0.001 | 0.000562169 | 0.820005 | 1 | 237.315 | 1039.11 | 1138 |
| 0.05 | 0.0095122 | 0.347405 | 1 | 5710.37 | 6036.94 | 20113 |
| 0.1 | 0.00528063 | 0.484673 | 1 | 5473.69 | 7791.9 | 32740 |
| 0.2 | 0.00746534 | 0.405593 | 1 | 9292.2 | 11106.3 | 52682 |
| 0.3 | 0.00947669 | 0.348315 | 1 | 12736.5 | 13490.4 | 68368 |
| 0.4 | 0.00715187 | 0.415694 | 1 | 13031.2 | 15916.9 | 81383 |
| 0.5 | 0.00450578 | 0.518742 | 1 | 11362.2 | 17520.3 | 92413 |
| 1 | 0.00349604 | 0.569932 | 1 | 12896 | 22592.6 | 123553 |

Table 7. PRS analysis for the nasal superior sector PRS for retinal thickness. See Table 1. for column titles explanations.

Nasal inferior sector PRS

| Threshold | R2 | P | Corrected P | Coefficient | Standard Error | No. of SNP |
| --- | --- | --- | --- | --- | --- | --- |
| 0.001 | 0.0133735 | 0.346128 | 1 | 790.942 | 833.949 | 1138 |
| 0.05 | 0.0209533 | 0.237172 | 1 | 5791.34 | 4857.9 | 20113 |
| 0.1 | 0.00862881 | 0.449864 | 1 | 4781.26 | 6292.42 | 32740 |
| 0.2 | 0.0123617 | 0.365202 | 1 | 8170.75 | 8965.68 | 52682 |
| 0.3 | 0.0133861 | 0.345901 | 1 | 10343.7 | 10901 | 68368 |
| 0.4 | 0.0162443 | 0.298679 | 1 | 13420.1 | 12818.4 | 81383 |
| 0.5 | 0.0175358 | 0.28004 | 1 | 15316.9 | 14071.1 | 92413 |
| 1 | 0.0129805 | 0.353374 | 1 | 16980.2 | 18176.4 | 123553 |

Table 8. PRS analysis for the nasal inferior sector PRS for retinal thickness. See Table 1. for column titles explanations.

**Power Calculation**


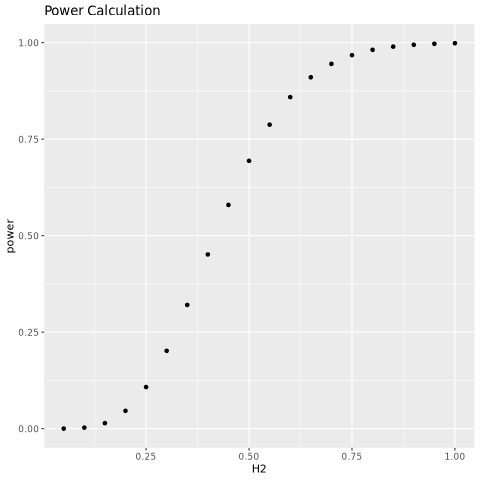


Figure 10. Power calculation. The y-axis has the statistical power and the x-axis (H2) is the trait variance.
